# A Mix and Match Approach to COVID-19 Vaccinations: Does Nigeria Have a Choice?

**DOI:** 10.3389/fpubh.2021.755974

**Published:** 2021-11-01

**Authors:** Ahmad Ibrahim Al-Mustapha, Muftau Oyewo, Abdullateef Saliman Olugbon, Nusirat Elelu

**Affiliations:** ^1^Department of Veterinary Services, Kwara State Ministry of Agriculture and Rural Development, Ilorin, Nigeria; ^2^Department of Veterinary Public Health and Preventive Medicine, Faculty of Veterinary Medicine, University of Ibadan, Ibadan, Nigeria; ^3^Faculty of Pharmaceutical Sciences, Universite de Tours, Tours, France; ^4^Nigeria Field Epidemiology and Laboratory Training Program, Asokoro, Nigeria; ^5^Department of Veterinary Public Health and Preventive Medicine, Faculty of Veterinary Medicine, University of Ilorin, Ilorin, Nigeria

**Keywords:** COVID-19, mix and match, vaccines, vaccine inequalities, Nigeria

The COVID-19 pandemic has caused severe global public health threats. To complement non-pharmaceutical interventions, vaccines were rapidly developed to save lives and curb the spread of the severe acute respiratory syndrome coronavirus 2 (SARS-CoV-2), the causative agent of COVID-19 (1).

In Nigeria, the emergence of the -more transmissible- delta SARS-CoV-2 variant (2), the increasing incidence of COVID-19 (Figure 1) (3), the poor public compliance with the COVID-19 non-pharmaceutical preventive practices such as the use of face masks, and the COVID-19 pandemic fatigue has further increased the need for COVID-19 vaccines (3). However, global economic realities resulted in inequalities in COVID-19 vaccine distribution as most high-income countries have fully vaccinated 49–61% of their population while low-and-middle-income countries (LMICs) in sub-Saharan Africa have only vaccinated 0.7% (in Nigeria) to 5.7% (in South Africa) of their population (4). For Instance, low-income countries would require an additional USD 38 billion to their 2021 GDP forecast if they wish to attain the same vaccination rate as high-income countries (5).

**Figure 1:**
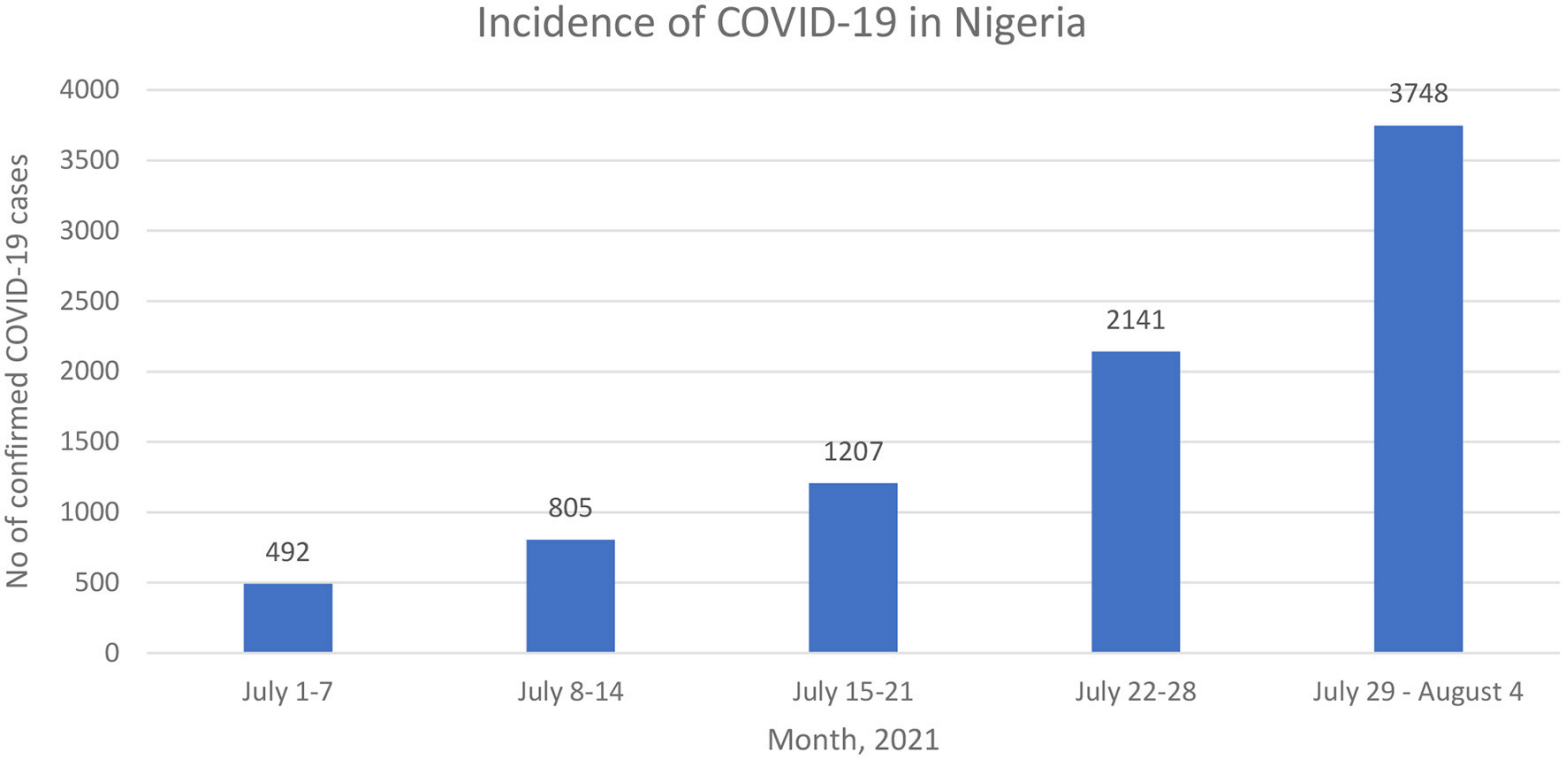
Weekly incidence of confirmed COVID-19 incidence in Nigeria (3).

To solve the problem of equitable global vaccine access especially in LMICs, the World Health Organization (WHO), the Global Alliance for Vaccines and Immunizations (GAVI), and the Coalition for epidemic Preparedness Innovations (CEPI) established the COVID vaccine facility (COVAX). COVAX has procured 2 billion COVID-19 vaccines from several pharmaceutical companies and periodically distributes them to 92 LMICs including Nigeria. Furthermore, several countries donate COVID-19 vaccines to LMICs such as Nigeria based on bilateral relations (6).

Nigeria's first COVID-19 vaccine (4 million doses of the Oxford-AstraZeneca vaccine) arrived on the 2nd of March 2021 from the COVAX facility (7). Nigeria prioritized frontline healthcare workers, elder statesmen, and initially persons older than 60 years to receive the vaccine. The first phase of vaccinations was concluded on the 22nd of July at which time 2,534,205 people who have been vaccinated for the first dose while some 1,404,205 Nigerians received the second dose of the vaccine. Hence, only 0.66% of Nigerians have been fully vaccinated (8). So, Nigeria currently has one of the worst COVID-19 vaccination rates in Sub-Saharan Africa (Table 1).

**Table 1:** Status of COVID-19 vaccinations in selected countries across the world as of 6th August 2021.

**Country**	**Income group (9)**	**Population (Million) (10)**	**No of the first dose administered (11)**	**% Fully vaccinated persons (11)**
Nigeria	Lower middle income	201	4 million	0.7% (1,404,205)
South Africa	Upper middle income	58.6	6.5 million	5.7% (3,327,950)
UK	High income	66.7	39,375,416	65.6% (33,093,568)
Morocco	Upper middle income	36.5	15,037,701	29.8% (10, 896,199)
Italy	High income	60.4	39, 517,945	54.8% (33,274,227)
Brazil	Upper middle income	211	108,567,088	21.4% (45,188,539)
Niger	Low income	23.3	363,118	0.3% (60,317)

Most LMICs including Nigeria do not produce any of the COVID-19 vaccines. So, in this regard, they are beggars with no choice. However, these countries have choices in their selection of the recipients for these different vaccines.

## Beggars Have No Choice

Low-and-middle-income countries such as Nigeria are reliant on international donations from high-income countries and the COVAX facility to access the COVID-19 vaccines for their citizenry. This is mostly due to the poor infrastructural capacity needed for vaccine production and development as well as ethical concerns associated with the transfer of patents. Aside from the ~4 million utilized doses of the Oxford-AstraZeneca vaccine, Nigeria has on the 1st of August received another 4 million doses of the Moderna vaccine as donations from the United State of America (12).

The Executive Director of the National Primary Health Care Agency (NPHCDA); Dr. Faisal Shuaibu announced that Nigeria is also expecting more COVID-19 vaccines. These vaccines are 3,924,000 doses of Oxford-AstraZeneca from the COVAX facility and 3,930,910 doses of Pfizer-Bio-N Tech COVID-19 vaccine from the COVAX facility donated by the U.S Government. In addition, Nigeria will get another 3.5 million doses of Pfizer-Bio-N Tech COVID-19 vaccine from the COVAX facility and 29,850,000 doses of Johnson & Johnson COVID-19 vaccine that will arrive in batches from the African Union Commission” (8).

Although most LMICs including Nigeria needs to scale up their COVID-19 vaccination rates and ensure that their citizens get the two doses of the vaccine, vital questions on the safety and efficacy of the mix and match approach remain unanswered, and caution must be taken by individuals as well as national public health agencies to ensure that the benefit of the mix-matched COVID-19 vaccination outweighs its risks. Furthermore, a transparent, holistic, and participatory approach by the Nigerian government is critical to combating the COVID-19 pandemic and rebuilding stronger health systems for its citizenry.

Currently, there are limited evidence on the safety, efficacy, and side effects of mix-matching COVID-19 vaccines. Hence, this viewpoint opines that until further evidence-based data is available, Nigerians who have taken the Oxford-AstraZeneca (viral-vector based) vaccine should not take the Moderna (mRNA based) COVID-19 vaccine.

## Mix- and Match Approach to COVID-19 Vaccines

Globally, severe logistical challenges have been experienced in the rapid, and global distribution of COVID-19 vaccines, especially in LMICs (5). These have resulted in the utilization of multiple types of COVID-19 vaccines since these vaccines induce immunity against SARS-CoV-2 via several approaches (RNA-based vaccines, subunit vaccines, inactivated virus, and virtual vector-based vaccines) (13). However, most of these vaccines require a second homologous booster dose. This has raised fundamental questions and challenges for LMICs such as Nigeria. These challenges centered around Nigeria's need to reduce the cost associated with vaccination, increasing vaccination acceptance, and the fluctuating supplies of vaccines. Furthermore, additional complexities due to differences in shelf life and storage conditions are associated with heterologous vaccines (different vaccine types) (13).

These challenges have made LMICs such as Nigeria accept any COVID-19 vaccine sent to them from different sources and will administer at least three different types of COVID-19 vaccines before the end of 2021 (8). This is called COVID-19 vaccine mix and match. Mix-matching of vaccines has been rationalized as safe, and an effective means to boost the immune response, and necessary to ensure the usage of currently available vaccines in LMICs (14).

Preliminary data from concluded and ongoing mix and match COVID-19 vaccination approach (13) showed that the strategy could enhance host immunity by preventing the development of viral immunity against a type of vaccine (13). In addition, the strategy will ensure that more people are vaccinated across the world and might be the magic wand needed to reduce the global inequalities in vaccine availability (13). Deming and Lyke (15) reported that the mix and match vaccination strategy improved the neutralization of variants (16) and the induction of spike protein-specific CD8+ T cells beyond what was achieved via homologous vaccinations (17). Furthermore, Barros-Martins et al. (16) and Schmidt et al. (17) believed that the heterologous combination of Oxford-AstraZeneca vaccine as primer dose and Pfizer–BioNTech Vaccine as booster dose resulted in 20 to >60-fold greater titers of neutralizing antibodies (IgG and IgA) against the alpha, beta, or gamma variants of the SARS-CoV-2. However, we believe that antibody and T-cell measurements in these studies do not correspond to real-life protection against the SARS-CoV-2 as these studies were only designed to assess the elicited immune responses after mixing vaccines (13).

Currently, only a few high-income countries (Canada, France, Germany, Denmark, Norway, Russia, South Korea, and Sweden) have advocated for the mixing of vaccines to their citizens in the light of rare thrombotic complications with the Oxford-AstraZeneca vaccine or due to its unavailability in their countries (18). The UK's rationale to adopt the mix and match approach was born out of the urgent need to vaccinate more citizens in the absence of the Oxford-AstraZeneca vaccine as its COVID-19 burden increases especially with the emergence of the Delta variant (B.1.617.2) (19).

However, the World Health Organization (WHO) opined that there is insufficient evidence to support the utilization of the mix and match approach and described it as a “dangerous trend” (20). The WHO therefore wanted more research conducted and evidence gathered before it could recommend mix and match for the COVID-19 vaccines. Some of the potential risk associated with mix-matching COVID-19 vaccines includes the regulatory complications and the possibility of increased adverse event following immunization (AEFI). During the first phase of the vaccinations using the Oxford-AstraZeneca vaccine, Nigeria recorded 14,550 cases of mild to moderate AEFI, with 148 cases considered to be severe (8).

In line with the recommendation of the WHO, the executive director of NPHCDA, Dr. Faisal Shuaibu recommended that Nigerians who had taken the first shot of the Oxford-AstraZeneca Vaccine should await the arrival of more Oxford-AstraZeneca vaccines (to be delivered to Nigeria in September) and not take the Moderna vaccine as the country plans a second phase of vaccinations (8).

While Nigeria and other LMICs race to vaccinate their citizens against COVID-19, this viewpoint opines that Nigeria's NPHCDA should only administer the Moderna COVID-19 vaccine to naïve recipients and not to those that have taken any shot of the Oxford-AstraZeneca vaccine. Furthermore, we strongly believe that whilst many countries may adopt the COVID-19 vaccine mixing strategy, Nigeria should not adopt the approach as Nigeria still has over 200 million unvaccinated citizens. So, Nigeria should ONLY use the Moderna vaccine for previously unvaccinated persons.

## Author Contributions

All authors listed have made a substantial, direct, and intellectual contribution to the work, and approved it for publication.

## Conflict of Interest

The authors declare that the research was conducted in the absence of any commercial or financial relationships that could be construed as a potential conflict of interest.

## Publisher's Note

All claims expressed in this article are solely those of the authors and do not necessarily represent those of their affiliated organizations, or those of the publisher, the editors and the reviewers. Any product that may be evaluated in this article, or claim that may be made by its manufacturer, is not guaranteed or endorsed by the publisher.
